# Protective Effect of Cactus Cladode Extracts on Peroxisomal Functions in Microglial BV-2 Cells Activated by Different Lipopolysaccharides

**DOI:** 10.3390/molecules22010102

**Published:** 2017-01-07

**Authors:** Fatima-Ezzahra Saih, Pierre Andreoletti, Stéphane Mandard, Norbert Latruffe, M’Hammed Saïd El Kebbaj, Gérard Lizard, Boubker Nasser, Mustapha Cherkaoui-Malki

**Affiliations:** 1 Laboratoire BioPeroxIL, Biochimie du Peroxysome, Inflammation et Métabolisme Lipidique, EA 7270, Unité de Formation et de Recherche des Sciences Vie, Terre et Environnement, 21000 Dijon, France; f.z.saih@hotmail.com (F.-E.S.); pierre.andreoletti@u-bourgogne.fr (P.A.); norbert.latruffe@u-bourgogne.fr (N.L.); gerard.lizard@u-bourgogne.fr (G.L.); 2 Laboratoire BioPeroxIL, Université Bourgogne-Franche Comté, 6 Bd Gabriel, 21000 Dijon, France; 3 Laboratoire de Biochimie et Neurosciences, Faculté des Sciences et Techniques, Université Hassan I, BP 577, 26 000 Settat, Morocco; boubker_nasser@hotmail.com; 4 Lipness Team, INSERM, Research Center UMR866 and LabEx LipSTIC, Faculté de Médecine, Université de Bourgogne-Franche Comté, 21079 Dijon CEDEX, France; stephane.mandard@u-bourgogne.fr; 5 Laboratoire Biologie et Santé, Faculté des Sciences Ben M’sik, Université Hassan II-Casablanca, Avenue Cdt Driss El Harti BP 7955, 20100 Casablanca, Morocco; mselkebbaj@yahoo.fr

**Keywords:** acyl-CoA oxidase 1, catalase, β-oxidation, *Escherichia coli*, lipopolysaccharides, LPS, nitric oxide, Opuntia, peroxisomes, *Salmonella minnesota*

## Abstract

In this study, we aimed to evaluate the antioxidant and anti-inflammatory properties of *Opuntia ficus-indica* cactus cladode extracts in microglia BV-2 cells. Inflammation associated with microglia activation in neuronal injury can be achieved by LPS exposure. Using four different structurally and biologically well-characterized LPS serotypes, we revealed a structure-related differential effect of LPS on fatty acid β-oxidation and antioxidant enzymes in peroxisomes: *Escherichia coli*-LPS decreased ACOX1 activity while *Salmonella minnesota*-LPS reduced only catalase activity. Different cactus cladode extracts showed an antioxidant effect through microglial catalase activity activation and an anti-inflammatory effect by reducing nitric oxide (NO) LPS-dependent production. These results suggest that cactus extracts may possess a neuroprotective activity through the induction of peroxisomal antioxidant activity and the inhibition of NO production by activated microglial cells.

## 1. Introduction

Excessive oxidative stress triggered by the generation of reactive oxygen species (ROS) has been linked to aging, neuroinflammation and neurodegenerative diseases such as Alzheimer disease, multiple sclerosis and peroxisomal leukodystrophies [1]. Microglia plays a central role in the neuroinflammation and in the physiopathogenesis of these disorders by producing proinflammatory molecules, such as tumor necrosis factor-α (TNF-α), interleukin-1 and 6, and nitric oxide (NO) [2,3,4]. Overproduction of ROS by activated microglia may affect healthy neurons thereby triggering neurodegeneration [5]. It has been reported that conditioned media induced microglia-damaged neurons [6] and that lipopolysaccharides (LPS)-activated microglia induced death of developing oligodendrocytes [7]. Furthermore, LPS activates microglia in the brain of animal models for sepsis [8]. Generation of ROS by LPS-activated microglia plays a key role in the triggered neurotoxicity [9], which can be largely reduced by neutralizing the extracellular hydrogen peroxide and superoxide by catalase and superoxyde dismutase treatment respectively [10,11]. Such results underlined the antioxidant role of peroxisomal proteins, particularly catalase.

Interestingly, peroxisomes are increasingly recognized as potential regulators of oxidative stress-related signaling pathways [12]. Hence, many peroxisomal enzymes catalyze redox reactions with the generation of hydrogen peroxide as a by-product. Excessive generation of ROS is known to disturb peroxisomal functions [13]. Peroxisome defect is associated with rare inborn errors of peroxisomal metabolism and peroxisomal leukodystrophies are part of neurodegenerative diseases with a progressive demyelination [1,14,15]. The origin of several of these leukodystrophies is linked to the absence of functional peroxisomal β-oxidation of very long chain fatty acids (VLCFA) due to the absence of one functional peroxisomal protein involved in the peroxisome biogenesis (i.e., Zellweger syndrome), in the transport or in the metabolism of VLCFA in the peroxisome (i.e., x-adrenoleukodystrophy and acyl-CoA oxidase 1 (ACOX1) deficiency) [1,14,15]. The absence of functional peroxisome caused progressive demyelination and recent data point out oxidative stress as the first hit in the development of neurodegeneration and demyelination [16].

Numerous data are supporting the beneficial role of dietary antioxidant and anti-inflammatory phytochemicals to reduce the deleterious effect of ROS and associated inflammatory processes related to neurodegenerative diseases [17]. *Opuntia ficus-indica*, commonly referred to as prickly pear or nopal cactus, is used in the sub-Saharan traditional medicine pharmacopeia and isolated compounds from its different aerial parts (cladodes, flowers and fruits) revealed antioxidant, anticancer, neuroprotective, hepatoprotective, and antiproliferative properties [17]. The cactus cladodes contain vitamins, antioxidants and various flavonoids, particularly quercetin 3-methyl ether, a highly efficient radical scavenger [18,19]. This supports the hypothesis that *Opuntia ficus indica* derived extracts might alleviate neuronal damages resulting or not from microglial activation.

**Figure 1:**
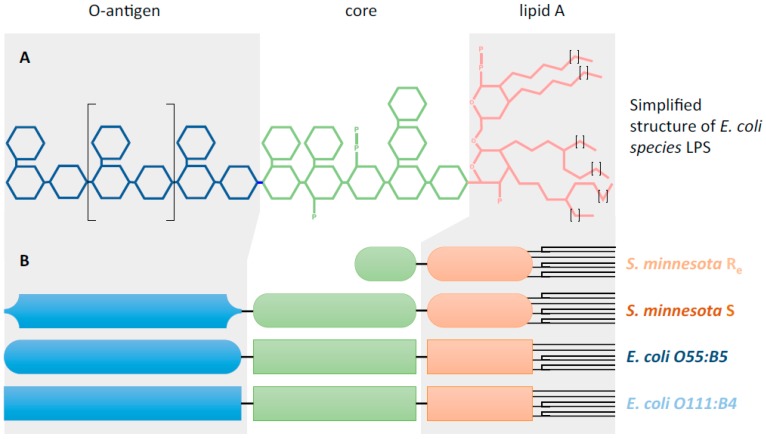
Structure of lipopolysaccharides. (**A**) Simplified structure of *E. coli* species LPS. LPS consist of three parts: (i) Lipid A, composed of two β1-6-linked *N*-acetylglucosamines (GlcNAc) substituted with six fatty acids (with chain length ranging from C12 to C16, buried in the bacterial outer membrane, marked with small brackets) as ether amino or hydoxyl linkage and with phosphate esters (“P”). Lipid A structure varies between bacterial strains in terms of fatty acids chain number, composition, phosphorylation and amination (not shown); (ii) the core, a polysaccharide moiety (composed of 3-deoxy-d-mannooctulosonic acid, heptose, glucose, galactose and GlcNAc with possible phosphorylation and amination), is linked to the lipid A; (iii) the O-antigen, composed of repetitive units of polysaccharides, defines the serotype of bacterial strains and can be composed of glucose, galactose, rhamnose, mannose or abequose. (**B**) Schematic structures of LPS used in this study. Selected LPS show different lengths or composition. Two LPS from *Salmonella minnesota* strains (*S. minnesota*) were used, one corresponds to the long form issued from the smooth strain (S); and the second one results from defects in LPS biosynthesis leading to truncated “rough” LPS chemotype, with the absence of O-antigen and a shortened core (*Re* form) (Huang et al., 2012). *E. coli* LPS differ in the O-antigen composition.

Previous reports related to the effect of LPS on peroxisome functions revealed that these endotoxins altered peroxisomal membrane composition, diminished the yield of peroxisome fraction in rat liver and enhanced peroxisomal proteins expression [20,21]. We have recently shown down-regulation of genes involved in hepatic peroxisomal fatty acid oxidation (FAOx) in mice exposed to LPS [22]. LPS strongly decreased the activity of ACOX1 and the oxidation of VLCFAs in rat C6 glial cells [23]. In ACOX1-deficient fibroblasts, LPS treatment induced the peroxisomes proliferation through the activation of PPARα and PGC-1α [24]. Intriguingly, data compilation regarding the nature of LPS used in the activation of microglial BV-2 cells revealed a great structural diversity from the published literature, whenever LPS serotype is clearly indicated in the related papers [4] (supplementary references). Recently, a systematic review of the in vivo experiments evidenced the disparity in timing and intensity of the LPS-dependent microglia reaction [25]. Such disparity in the generated data can be explained, in all likelihood, by the structural dissimilarity of LPS used in these studies for microglia activation. Thus, the misinterpretation of results can be increased by the great variation in the used conditions of cell culture and LPS serotype. LPS is derived from the gram-negative bacteria, in which it is an integral component of the outer leaflet of the outer membrane [26]. Figure 1A presents simplified structures of enterobacteria LPS. Indeed, LPS consist of three parts: (i) The lipid A, composed of two β1-6-linked *N*-acetylglucosamines (GlcNAc) substituted with six fatty acids (with chain length ranging from C12 to C16, buried in the bacterial outer membrane) as ether amino or hydroxyl linkage and with phosphate esters. Lipid A structure varies between bacterial strains in terms of fatty acids chain number, composition, phosphorylation and amination; (ii) the core, a polysaccharide moiety (composed of 3-deoxy-d-mannooctulosonic acid, heptose, glucose, galactose and GlcNAc with possible phosphorylation and amination), linked to the lipid A; (iii) and the O-antigen, composed of repetitive units of polysaccharides, defines the serotype of bacterial strains and can be composed of glucose, galactose, rhamnose, mannose or abequose [26].

In the present work, we investigated the antioxidant and anti-inflammatory effects of *Opuntia ficus-indica* cactus cladode extracts in LPS-activated microglia BV-2 cells. Four different LPS serotypes with different lengths and structures (Figure 1B) were used to activate BV-2 cells [26,27,28]: two *E. coli* LPS differ only in the O-antigen chemical composition (Figure 1B); and two LPS from *Salmonella minnesota* strains (*S. minnesota*), one corresponds to the long form issued from the smooth strain (S) and the second one results from defects in LPS biosynthesis leading to truncated “rough” LPS chemotype, with the absence of O-antigen and a shortened core (*Re* form) [28]. The antioxidant activity of the different cladode extracts was evaluated through the measurement of catalase and ACOX1 activities, aiming to specify the preventive effect of cactus extracts on peroxisomal dysfunction during LPS-dependent microglia activation, while the anti-inflammatory activity was tested with NO production by BV-2 cells.

## 2. Materials and Methods

### 2.1. Chemicals

LPS were obtained from Sigma-Aldrich, St. Quentin Fallavier, France (*Escherichia coli O55:B5*, L2880; *E. coli O111:B4*, L2630) and from Enzo Life Sciences, Villeurbanne, France (*Salmonella minnesota* S-Form, ALX-581-020; *S. minnesota* R595 (Re), ALX-581-008).

### 2.2. BV-2 Microglia Cell Culture

Murine microglial BV-2 cell lines (BV-2) were grown in a 5% CO_2_ incubator at 37 °C in Dulbecco’s modified Eagle medium (DMEM) supplemented with 10% (*v*/*v*) heat inactivated fetal bovine serum (FBS) and 1% antibiotics (penicillin, streptomycin); culture medium was changed every 2 days. BV-2 cells were seeded on 6-well microplates at 5 × 10^5^ cells/well for viability assay, 96-well microplates at 2 × 10^4^ cells/well for the NO assay, 24-well microplates at 1 × 10^5^ cells/well for catalase activity and Western blotting analysis. Cells were treated with 10, 30 or 100 µg/mL of cactus cladodes extracts and LPS at 1 µg/mL dissolved in fresh DMEM with 5% of FBS.

### 2.3. Cactus Extracts Preparation

Four cactus cladode extracts were prepared with solvents of increasing polarity using hexane and chloroform as non-polar solvents and ethylacetate and methanol as polar solvents [29,30]. Forty milligrams of cladodes from *Opuntia ficus-indica* plants were dried at 50 °C for 48 h, grinded, macerated with methanol 80% for 48 h and filtered. Filtrate was then successively extracted with hexane, chloroform, ethylacetate and finally methanol. The four extracts were evaporated and residues were dissolved in DMSO 50% and kept at 4 °C in darkness up to use.

### 2.4. MTT Assay

The cell proliferation and/or mitochondrial activity were measured using MTT (3-(4,5-dimethyltrazol-2-yl)-2,5-diphenyltetrazolium bromide) assay [31]. Cells, plated in 6-wells plates, were treated for 24 h with the four different serotypes of LPS (1 or 2 µg/mL) or the four cactus extracts (100 µg/mL). Cells were incubated for 2 h with MTT dye followed by the absorbance (Abs) measurement at the 570 nm with a microplate reader.

### 2.5. Staining with Crystal Violet Assay

Quantification of the adherent cell was estimated by staining with crystal violet [32]. Cells were seeded in 6-wells plates and treated for 24 h with the four different serotypes of LPS (1 or 2 µg/mL) or the four cactus extracts (100 µg/mL). At the end of treatment, cells were washed with phosphate buffer saline, stained with crystal violet, rinsed with water and after methanol addition, the optical density (OD) was measured at the 570 nm with a microplate reader.

### 2.6. Nitrite Assay

The production of NO was determined by measuring nitrite (NO_2_^−^) accumulation in the cell culture media. BV-2 cells were pretreated with the four different cactus cladodes extracts at 10, 30 or 100 µg/mL for 4 h and then stimulated with 1 µg/mL LPS for 24 h. The accumulated nitrite in the culture supernatant, used as an indicator of NO production, was measured using the Greiss reaction method [33]. Each supernatant was mixed with an equal volume of the Greiss reagent (Sigma-Aldrich) and the absorbance of the mixture was measured at the 540 nm with a microplate reader.

### 2.7. Preparation of BV-2 Cell Lysate

After treatment of BV-2 microglia cells with the four different cactus cladodes extracts and with LPS for 24 h, cells were washed with PBS buffer and 1 × 10^5^ cells were lysed in 50 µL of Radioimmunoprecipitation assay (RIPA) buffer: 50 mM Tris-HCl, pH 7.4, 1% NP-40, 0.5% Na-deoxycholate, 0.1% sodium dodecylsulfate, 150 mM NaCl, 2 mM Ethylenediamine tetraacetic acid (EDTA), 50 mM NaF. Cells were placed in ice for 30 min and the lysate was cleared by centrifugation at 20,000× *g* for 20 min. Protein content was assessed by a bicinchoninic acid assay [34]. The supernatant was stored at −80 °C until further use.

### 2.8. Enzymatic Activity Measurement

For catalase activity measurement, 10 µL of BV-2 cell lysate was added to 190 µL of Tris HCl buffer (pH = 7.4) containing 20 mM H_2_O_2_ and the decrease of the absorbance was monitored at 240 nm for 2 min [35]. The change in absorbance with time was proportional to the breakdown of H_2_O_2_. The catalase activity was expressed as units/mg of protein. ACOX1 activity measurement was performed as described by Oaxaca-Castillo D. et al. [36].

### 2.9. Western Blotting Analysis

The BV-2 microglia were pretreated with the four different cactus cladodes extracts at 10, 30 or 100 µg/mL for 4 h and then stimulated with LPS at 1 µg/mL for 24 h; the cells were lysed in lysis buffer. For Western blotting analysis, 30 µg of protein was separated by 15% SDS-page and transferred onto polyvinylidne difluoride (PVDF) membranes [37]. Membranes were blocked using blocking buffer Tris-buffered Saline buffer containing 5% milk, 0.1% Tween 20 (TBST) and washed three times with TBST 5 min each. Membranes were incubated with rabbit anti-catalase antibody (1:1500) in 5% milk in TBST at room temperature for 3 h, washed three times with TBST, incubated with a goat anti-rabbit secondary antibody coupled to horseradish peroxidase for 1 h and developed using enhanced chemiluminescence (ECL) Western blotting detection (Santa Cruz Biothechnology, Inc., Heidelberg, Germany).

### 2.10. Statistical Analysis

Statistical analyses to compare two experimental groups were performed with an unpaired, two-tailed, Student-t test (Excel software) for calculating the probability values; and data were considered statistically different at a *p*-value of 0.05 or less.

## 3. Results

### 3.1. Effect of Different LPS on Mitochondrial Status and Viability of Microglial BV-2 Cells

**Figure 2:**
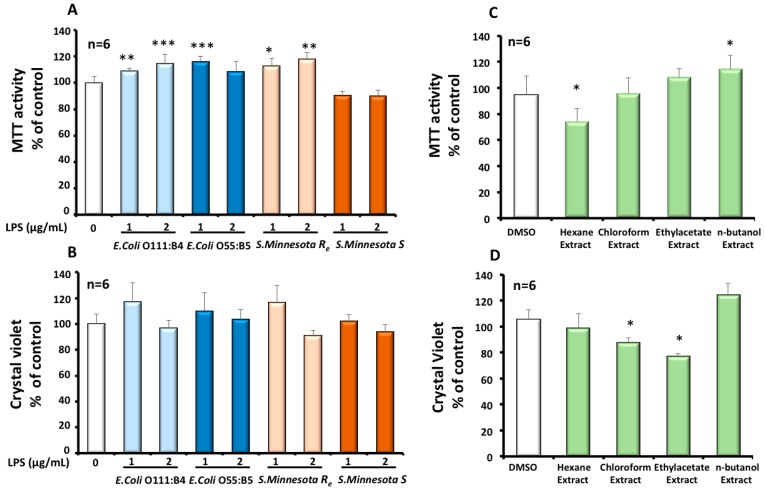
Effect of LPS or cactus extracts on viability (MTT assay; **A** and **C** respectively) and on cell growth (crystal violet assay; **B** and **D** respectively) of BV-2 microglial cells. Cells were treated for 24 h with the four different serotypes of LPS (1 or 2 µg/mL) or the four cactus extracts (100 µg/mL). The results were the mean ± SD of the three independent experiments. Values were normalized to the control and are given as percent of the control. The significance is shown with the Student-t test: *p* < 0.05 for *; *p* < 0.01 for **; *p* < 0.001 for ***.

A MTT test was performed to estimate the effect of different LPS on mitochondrial function and BV-2 cells viability. The amount of formazan produced is proportional to the number of metabolically active viable cells and involves the transfer of electrons to MTT by reducing molecules, such as NADH [38]. Both LPS, at 1 or 2 µg/mL, from *E. coli*, showed after 24 h an increase in the mitochondrial dehydrogenases activity. Only the smallest LPS from rough *S. minnesota Re*, with less oligosaccharides units on the core region and missing the O-antigen part, enhanced the activity of mitochondrial dehydrogenases (Figure 2A) after 24 h at the used concentrations of 1 or 2 µg/mL. A MTT assay also reflects cell viability and proliferation under different LPS treatments. Regarding the obtained results, we can conclude that LPS from both *E. coli* and *S. minnesota Re* were more prone to increase by 10% to 20% BV-2 cells viability and proliferation (Figure 2A). BV-2 microglial cells are reputed semi-adherent cells in DMEM medium. As shown by a crystal violet assay (Figure 2B), treatment with structurally different LPS had no significant effect on BV1 cell adhesion. This observation seems to be coherent with the obtained results for the MTT test.

### 3.2. Effects of Cactus Cladode Extracts on Mitochondrial Status and Viability of Microglial BV-2 Cells

A MTT test was performed to estimate the effect of the four cactus extracts on mitochondrial function and BV-2 cell viability. As shown in Figure 2C, the hexane extract and n-butanol extract have an opposite moderate effect on BV-2 cell viability. The former decreased the viability by −30%, while the latest increased BV-2 viability by +18%. On the other hand, using the crystal violet test, we showed that only chloroform and ethyl-acetate extracts have a negative effect on growth and adherence of BV-2 cells (Figure 2D). These negative effects were estimated to be −15% for the chloroform extract and to be −20% for and the ethyl-acetate extract respectively.

### 3.3. Effects of Different LPS on Peroxisomal Functions in Microglial BV-2 Cells

**Figure 3:**
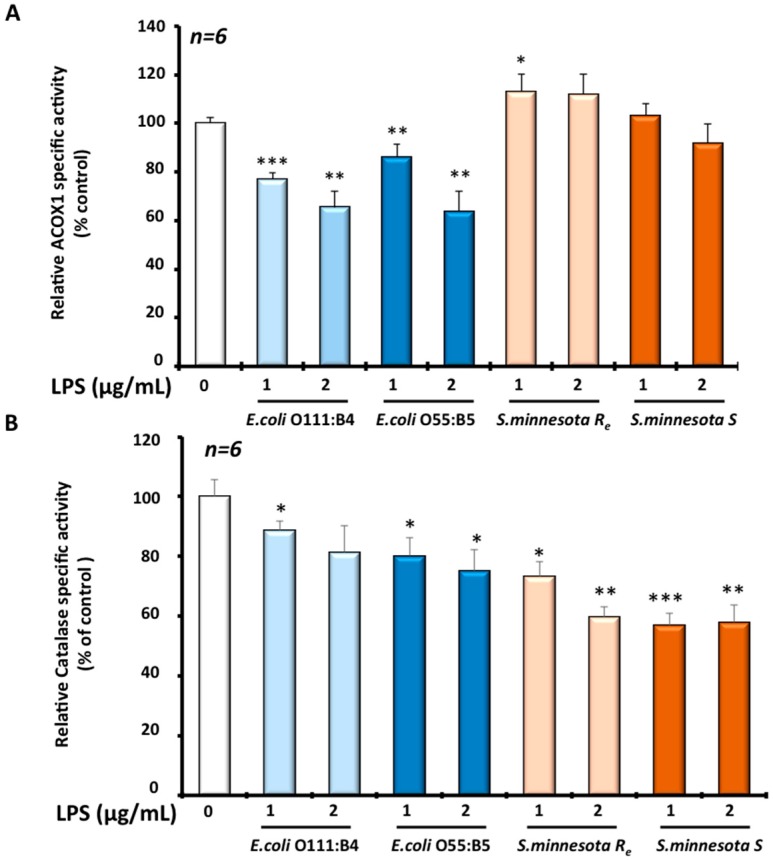
Enzymatic specific activities of (**A**) ACOX1 and (**B**) catalase were measured in BV-2 microglial cells treated for 24 h with the four different serotypes of LPS (1 or 2 µg/mL). The results were the mean ± SD of the three independent experiments. Values were normalized to the control and are given as percent of the control. The statistical significance was calculated with the Student-t test: *p* < 0.05 for *; *p* < 0.01 for **; *p* < 0.001 for ***.

**Figure 4:**
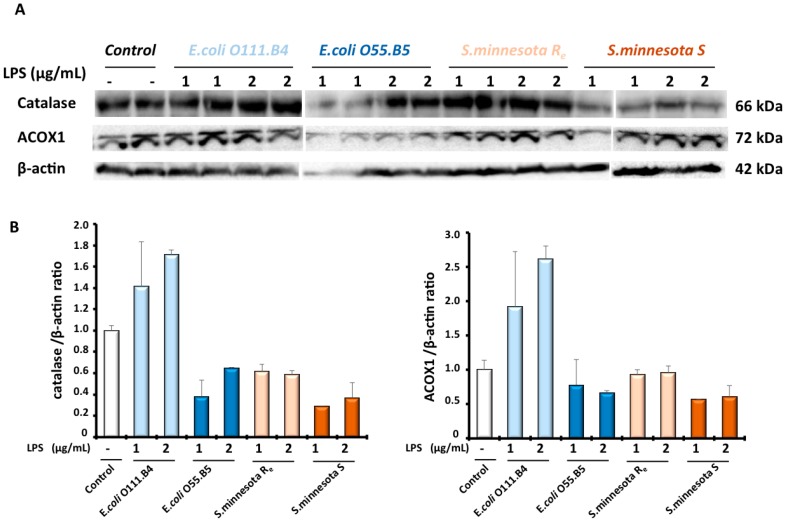
Expression levels of ACOX1 and catalase proteins were evaluated by Western blotting analysis in BV-2 cell extracts after LPS treatment with the four different serotypes (1–2 µg/mL) for 24 h (**A**). Expression of protein was quantified by densitometry analysis and normalized to the β-actin; (**B**) and values represent the fold induction relative to the control.

Peroxisome organelle is characterized by the presence of more than a dozen oxidase-producing H_2_O_2_ as a by-product and also catalase as an H_2_O_2_-degrading enzyme. Hence, we measured in BV-2 cells, after LPS treatment, the activity of ACOX1, the rate-limiting enzyme of peroxisomal β-oxidation, and the activity of catalase, the peroxisomal antioxidant enzyme. Intriguingly, results showed that different LPS impact ACOX1 activity differentially and catalase activity. Thus, both *E. coli* serotypes, *O111* and *O55* respectively, showed a dose-dependent decrease of ACOX1 activity in BV-2 cells after 24 h LPS treatment. ACOX1 activity was decreased by −25% to −35% with *O111 E. coli* serotype and −18% to −35% for *O55 E. coli* serotype respectively. *S. minnesota* serotypes generally had a slight (+13%) or no effect on ACOX1 activity (Figure 3A). By contrast, catalase activity was clearly decreased with both *S. minnesota* serotypes treatments. In the presence (*S*) of O-antigen, the catalase activity was decreased by −25% to −40%, relative to the control, while the absence of the O-antigen (*Re*) revealed a 43% decrease of catalase activity. However, *E. coli* serotypes treatment exhibited a significant but a relatively slight decrease estimated to be −12% to −20% for *O111 E. coli* serotype and −20% to −25% for *O55 E. coli* serotype (Figure 3B). Thus, regarding the effect different LPS on peroxisome functions, the effect of LPS on peroxisomal antioxidant function were inversely proportional to the effect on the activity of peroxisomal β-oxidation in BV-2 microglial cells. The protein levels of ACOX1 and catalase were evaluated by Western blotting. As shown in Figure 4, except *E. coli O111* serotype, which increases the catalase level by 1.4 to 1.7 fold, all the LPS serotypes decreased the level of catalase (0.3 to 0.6 fold) in BV-2 cells. ACOX1 level was only slightly decreased by different LPS, except *E. coli O111* serotypes for which we can see an increased expression of ACOX1 (2 to 2.6 fold) as for catalase (Figure 4).

### 3.4. Effects of Cactus Cladode Extracts on Catalase Expression in Microglial BV-2 Cells

**Figure 5:**
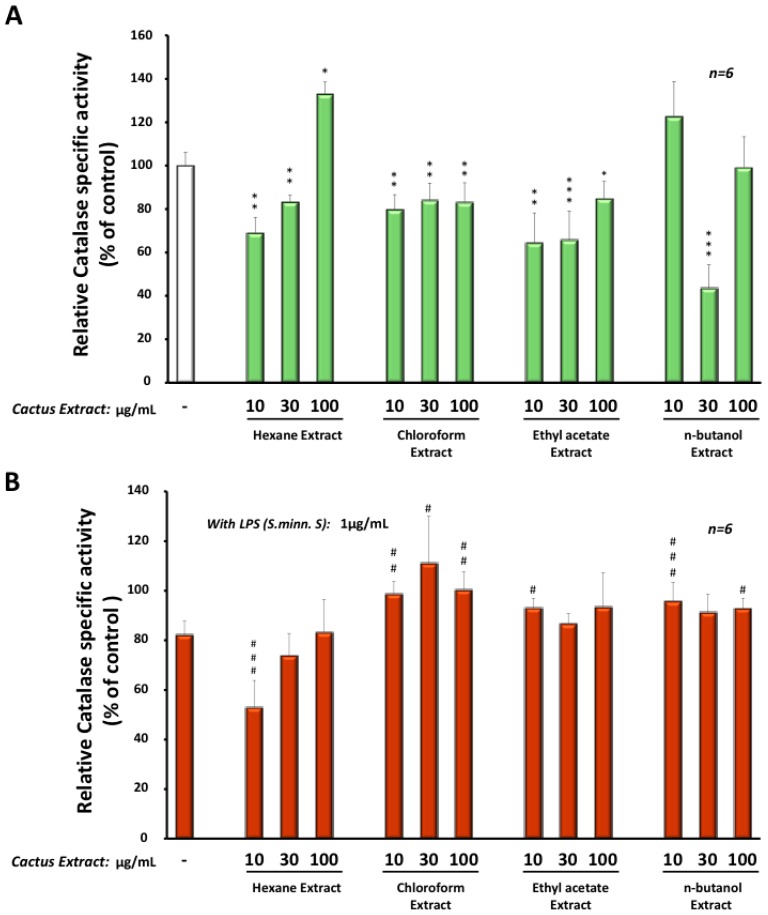
Enzymatic specific activity of catalase was measured in BV-2 microglial cells treated for 24 h with the four different cactus extracts (10, 30 or 100 µg/mL) without (**A**) or in the presence of *Salmonella minnesota S* LPS at 1 µg/mL (**B**). The results were the mean ± SD of the three independent experiments. Values were normalized to the control and are given as percent of the control. The statistical significance was calculated with the Student-t test: *p* < 0.05 for * or ^#^; *p* < 0.01 for ** or ^##^; *p* < 0.001 for *** or ^###^.

Intriguingly, extracts obtained with hexane, chloroform or ethyl-acetate decreased significantly the catalase activity between −20% and −55% in BV-2 cells after 24 h treatment, except for the hexane extract at the concentration of 100 µg/mL, showing an increase of 30% (Figure 5A). In the presence of LPS (from *Salmonella minnesota S* serotype) (Figure 5B), low dose, at 10 µg/mL, of the hexane extract, we observed a greater decrease (−45%) in catalase activity when compared to the LPS treatment alone. However, higher concentrations of the hexane extract or different doses of the other *Opuntia* extracts (chloroform, ethyl-acetate or *n*-butanol) reestablished catalase activity at the same or higher level than the control (Figure 5B). Interestingly, treatment with different extracts had no significant effect on catalase at the protein level (data not shown). This indicates that changes in catalase activity were the result of the modulation of the enzymatic activity. 

### 3.5. Anti-Inflammatory Effect of Cactus Cladode Extracts on NO Production in Microglial BV-2 Cells

**Figure 6:**
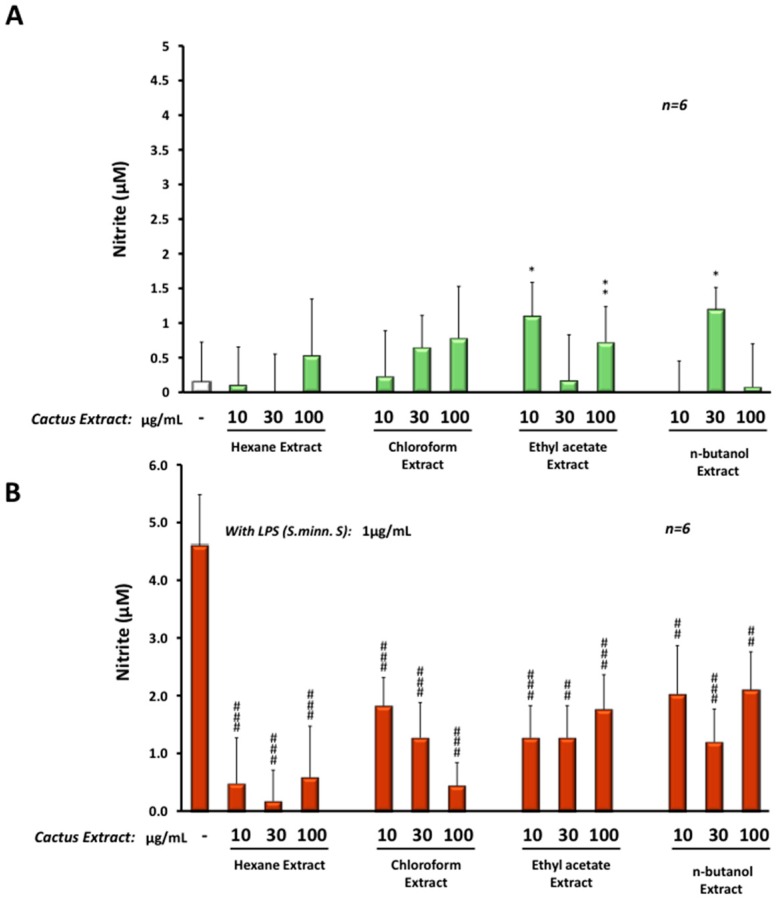
NO production (in µM) by BV-2 cells after 24 h treatment with the four different cactus extracts (10, 30 or 100 µg/mL) without (**A**) or in the presence of *Salmonella minnesota S* LPS at 1 µg/mL (**B**) evaluated by the Greiss test (NO production was determined by measuring nitrite (NO_2_^−^) accumulation in the cell culture media). The significance is shown with the Student-t test: *p* < 0.05 for *; *p* < 0.01 for ** or ^##^; *p* < 0.001 for ^###^.

To evaluate the anti-inflammatory protective effect of these cactus extracts, we treated microglial BV-2 cells with different extracts at three concentrations: 10, 30 or 100 µg/mL (using DMSO as vehicle) in the presence or absence of LPS from *Salmonella minnesota S* serotype. This serotype was chosen because of its significant effect on catalase activity. After 24 h of treatment, different cactus extracts induced a very slight production (up to 1 µM) of NO when compared to the control (Figure 6A). All extracts reduced the effect of LPS (24 h treatment at 1 µg/mL) on NO production in BV-2 cells, from 4.7 µM under LPS treatment alone to less than 2 µM in the presence of the cactus extract. However, hexane and chloroform extracts were the most efficient in reduction of NO production induced by LPS, with less than 1 µM of NO in a dose-dependent manner (Figure 6B).

## 4. Discussion

In this study, the antioxidant and anti-inflammatory properties of *Opuntia ficus-indica* cactus cladode extracts were evaluated in microglia BV-2 cells. Different LPS were used to achieve BV-2 cell activation because of the structural heterogeneity of the used LPS serotypes in published studies, whenever it was specified. Therefore, we decided to compare four different serotypes of LPS, which were structurally and biologically well defined [26,27,28] and presenting a specific structural feature in one of their three LPS parts (i.e., O-antigen, core and Lipid A). The *E. coli* and *S. minnesota S* LPS serotypes differ only in the O-antigen part, while *S. Minnesota Re* have no O-antigen and show shorter oligosaccharidic chain length in the core part (Figure 1); the lipid A part is hexacetylated in *E. coli* serotypes, while *S. minnesota* serotypes have an heptacetylated lipid A. We showed that three serotypes revealed only a slight increase (10% to 15%) of BV-2 cells viability, except for *S. minnesota S* serotype. Previous studies have shown a slight or no change in the viability of BV-2 cells after treatment with *E. coli O111:B4* chemotype [39]. Compared to the BV-2 cell line, primary microglia cells (isolated from rat or mouse newborn brains) are more sensitive to this chemotype and show a significant reduction of their viability as earlier as 2 h following LPS treatment [40,41]. For the *E. coli O55:B5* chemotype, reported results have shown no significant effect on BV-2 cell viability, but a decrease of primary microglia viability [42,43]. To our knowledge, the effect of Rough chemotypes of *S. minnesota* on BV-2 cells has not been described yet. However, in our study, only the *Re S. minnesota* chemotype induced a slight increase in BV-2 cell viability.

Here, we showed, for the first time, a differential effect of LPS between two peroxisomal activities (i.e., ACOX1 and catalase activities) in microglia BV-2 cells. Indeed, both *E. coli* serotypes *0111:B4* and *O55:B5* reduced ACOX1 activity (−20% to −40%) but only slightly catalase activity (−10% to −20%). However, BV-2 treatment with *S. minnesota* serotypes revealed a unique effect only on catalase activity (−40%) and no effect on ACOX1 activity. It should be noted that lipid A (from *E. coli*) treatment had no effect on ACOX1 activity (data not shown) indicating that the observed effect is not linked to the lipid A moiety itself. In addition, the expression levels of both ACOX1 and catalase proteins were reduced by all LPS except for *E. coli O55:B5* serotypes. An earlier report from Khan et al. has shown that the *E. coli* LPS inhibits the oxidation of VLCFA, leading to their accumulation in C6 glial cells [44]. Later, the same group showed that the *E. coli 055:B5* chemotype reduced drastically the β-oxidation of VLCFA as well as the expression of ACOX1 and catalase transcripts in the fetal rat brain after maternal LPS exposure [45]. Accordingly, endotoxin treatment deeply impacts liver peroxisomes in a specific manner as other cellular organelles are less affected or not affected. Indeed, Khan M. et al. showed that LPS from *Salmonella typhimurium* (structurally resembling the used *E. coli* serotype in this study) augmented the ratio of cholesterol/phospholipids, the plasmalogen levels and affected the fatty acid composition of the peroxisomal fraction [46]. The mechanism underlying this specific remodeling of peroxisomal lipids is still unknown. Our result showing the LPS-associated reduction of the peroxisomal ACOX1 activity may explain, at least partially, the perturbation of hepatic peroxisome lipid structure and metabolism under LPS treatment, which seems to be completely mediated by Kupffer cells, as liver resident macrophages [46]. Thus, the suppression of ACOX1 activity in microglial cells and its consequence on the peroxisomal capacity in the β-oxidation of VLCFA may have a deleterious effect on brain functions and must be associated to neuroinflammation and neurodegeneration in peroxisomal leukodystrophies, including ACOX1 deficiency [47]. This means that exposure of patients with peroxisomal disorders to bacterial infection may worsen their metabolic dysregulation.

In an attempt to explore the therapeutic potentialities of cactus extracts, we showed here that *Opuntia* cladode extracts prevent LPS-associated catalase activity decrease. This modulation of catalase activity was at the level of the enzymatic activity. It has been shown that a 90-kDa glycoprotein isolated from *Opuntia ficus indica* augmented catalase activity in mice liver [48]. Interestingly, different cactus extracts used in this study revealed a strong preventive effect on the production of NO by LPS in BV-2 cells. Our results underline the capacity of *Opuntia* cladode extracts to suppress the inflammatory response induced by LPS in microglial cells, as reported for other cactus tissue extracts [49]. In addition, in cultured mouse cortical cells, the *Opuntia* fruit extract significantly decreased delayed neurotoxicity induced by *N*-methyl-d-aspartate-, kainate-, and oxygen–glucose deprivation [50]. Thus, *Opuntia ficus indica* extracts might alleviate neuronal damages resulting from microglial activation.

Collectively, in this study, we showed that cladode extracts from *Opuntia ficus indica* revealed an antioxidant capacity in the modulation of peroxisomal catalase activity and clear anti-inflammatory properties. In addition, the regulation of peroxisomal functions, regarding LPS exposure, is clearly dependent on LPS chemotype; and LPS shape promotes differential responses between the peroxisomal fatty acid β-oxidation and the peroxisomal antioxidant function.

## References

[1] Trompier D., Vejux A., Zarrouk A., Gondcaille C., Geillon F., Nury T., Savary S., Lizard G. (2014). Brain peroxisomes. Biochimie.

[2] Pawate S., Shen Q., Fan F., Bhat N.R. (2004). Redox regulation of glial inflammatory response to lipopolysaccharide and interferongamma. J. Neurosci. Res..

[3] Perry V.H., Teeling J. (2013). Microglia and macrophages of the central nervous system: The contribution of microglia priming and systemic inflammation to chronic neurodegeneration. Semin. Immunopathol..

[4] Cunningham C. (2013). Microglia and neurodegeneration: The role of systemic inflammation. Glia.

[5] Von Bernhardi R., Eugenin-von Bernhardi L., Eugenin J. (2015). Microglial cell dysregulation in brain aging and neurodegeneration. Front. Aging Neurosci..

[6] Block M.L., Hong J.S. (2007). Chronic microglial activation and progressive dopaminergic neurotoxicity. Biochem. Soc. Trans..

[7] Li J., Baud O., Vartanian T., Volpe J.J., Rosenberg P.A. (2005). Peroxynitrite generated by inducible nitric oxide synthase and NADPH oxidase mediates microglial toxicity to oligodendrocytes. Proc. Natl. Acad. Sci. USA.

[8] Konsman J.P., Parnet P., Dantzer R. (2002). Cytokine-induced sickness behaviour: Mechanisms and implications. Trends Neurosci..

[9] Liu B., Du L., Hong J.S. (2000). Naloxone protects rat dopaminergic neurons against inflammatory damage through inhibition of microglia activation and superoxide generation. J. Pharmacol. Exp. Ther..

[10] Wang T., Liu B., Qin L., Wilson B., Hong J.S. (2004). Protective effect of the SOD/catalase mimetic MnTMPyP on inflammation-mediated dopaminergic neurodegeneration in mesencephalic neuronal-glial cultures. J. Neuroimmunol..

[11] Nell H.J., Au J.L., Giordano C.R., Terlecky S.R., Walton P.A., Whitehead S.N., Cechetto D.F. (2016). The targeted antioxidant, catalase-SKL, reduces beta-amyloid toxicity in the rat brain. Brain Pathol..

[12] Nordgren M., Fransen M. (2014). Peroxisomal metabolism and oxidative stress. Biochimie.

[13] Schrader M., Fahimi H.D. (2006). Peroxisomes and oxidative stress. Biochim. Biophys. Acta.

[14] Wanders R.J. (2014). Metabolic functions of peroxisomes in health and disease. Biochimie.

[15] Wanders R.J., Waterham H.R. (2006). Peroxisomal disorders: The single peroxisomal enzyme deficiencies. Biochim. Biophys. Acta.

[16] Singh I., Pujol A. (2010). Pathomechanisms underlying X-adrenoleukodystrophy: A three-hit hypothesis. Brain Pathol..

[17] El-Mostafa K., El Kharrassi Y., Badreddine A., Andreoletti P., Vamecq J., El Kebbaj M.S., Latruffe N., Lizard G., Nasser B., Cherkaoui-Malki M. (2014). Nopal cactus (Opuntia ficus-indica) as a source of bioactive compounds for nutrition, health and disease. Molecules.

[18] Lee J.C., Kim H.R., Kim J., Jang Y.S. (2002). Antioxidant property of an ethanol extract of the stem of Opuntia ficus-indica var. saboten. J. Agric. Food Chem..

[19] Stintzing F.C., Carle R. (2005). Cactus stems (Opuntia spp.): A review on their chemistry, technology, and uses. Mol. Nutr. Food Res..

[20] Contreras M.A., Khan M., Smith B.T., Cimini A.M., Gilg A.G., Orak J., Singh I., Singh A.K. (2000). Endotoxin induces structure-function alterations of rat liver peroxisomes: Kupffer cells released factors as possible modulators. Hepatology.

[21] Dhaunsi G.S., Hanevold C.D., Singh I. (1994). Impairment of peroxisomal beta-oxidation system by endotoxin treatment. Mol. Cell. Biochem..

[22] El Kebbaj R., Andreoletti P., El Hajj H.I., El Kharrassi Y., Vamecq J., Mandard S., Saih F.-E., Latruffe N., El Kebbaj M.S., Lizard G. (2015). Argan oil prevents down-regulation induced by endotoxin on liver fatty acid oxidation and gluconeogenesis and on peroxisome proliferator-activated receptor gamma coactivator-1α, (PGC-1α), peroxisome proliferator-activated receptor α (PPARα) and estrogen related receptor α (ERRα). Biochim. Open.

[23] Paintlia M.K., Paintlia A.S., Khan M., Singh I., Singh A.K. (2008). Modulation of peroxisome proliferator-activated receptor-alpha activity by N-acetyl cysteine attenuates inhibition of oligodendrocyte development in lipopolysaccharide stimulated mixed glial cultures. J. Neurochem..

[24] El Kebbaj R., El Kamouni S., El Hajj H.I., Andreoletti P., Gresti J., Latruffe N., El Kebbaj M.S., Vamecq J., Lizard G., Nasser B. (2013). Modulation of peroxisomes abundance by argan oil and lipopolysaccharides in acyl-CoA oxidase 1-deficient fibroblasts. Health.

[25] Hoogland I.C., Houbolt C., van Westerloo D.J., van Gool W.A., van de Beek D. (2015). Systemic inflammation and microglial activation: Systematic review of animal experiments. J. Neuroinflamm..

[26] Stenutz R., Weintraub A., Widmalm G. (2006). The structures of *Escherichia coli* O-polysaccharide antigens. FEMS Microbiol. Rev..

[27] Matsuura M. (2013). Structural Modifications of Bacterial Lipopolysaccharide that Facilitate Gram-Negative Bacteria Evasion of Host Innate Immunity. Front. Immunol..

[28] Huang J.X., Azad M.A., Yuriev E., Baker M.A., Nation R.L., Li J., Cooper M.A., Velkov T. (2012). Molecular Characterization of Lipopolysaccharide Binding to Human alpha-1-Acid Glycoprotein. J. Lipids.

[29] Sanchez E., Garcia S., Heredia N. (2010). Extracts of edible and medicinal plants damage membranes of Vibrio cholerae. Appl. Environ. Microbiol..

[30] Ennouri M., Ammar I., Khemakhem B., Attia H. (2014). Chemical composition and antibacterial activity of Opuntia ficus-indica f. inermis (cactus pear) flowers. J. Med. Food.

[31] Denizot F., Lang R. (1986). Rapid colorimetric assay for cell growth and survival. Modifications to the tetrazolium dye procedure giving improved sensitivity and reliability. J. Immunol. Methods.

[32] Badreddine A., Karym E.M., Zarrouk A., Nury T., Kharrassi Y.E., Nasser B., Cherkaoui-Malki M., Lizard G., Samadi M. (2015). An expeditious synthesis of spinasterol and schottenol, two phytosterols present in argan oil and in cactus pear seed oil, and evaluation of their biological activities on cells of the central nervous system. Steroids.

[33] Green L.C., Wagner D.A., Glogowski J., Skipper P.L., Wishnok J.S., Tannenbaum S.R. (1982). Analysis of nitrate, nitrite, and [^15^N]nitrate in biological fluids. Anal. Biochem..

[34] Smith P.K., Krohn R.I., Hermanson G.T., Mallia A.K., Gartner F.H., Provenzano M.D., Fujimoto E.K., Goeke N.M., Olson B.J., Klenk D.C. (1985). Measurement of protein using bicinchoninic acid. Anal. Biochem..

[35] Cherkaoui-Malki M., Bardot O., Lhuguenot J.C., Latruffe N. (1990). Expression of liver peroxisomal proteins as compared to other organelle marker enzymes in rats treated with hypolipidemic agents. Biol. Cell.

[36] Oaxaca-Castillo D., Andreoletti P., Vluggens A., Yu S., van Veldhoven P.P., Reddy J.K., Cherkaoui-Malki M. (2007). Biochemical characterization of two functional human liver acyl-CoA oxidase isoforms 1a and 1b encoded by a single gene. Biochem. Biophys. Res. Commun..

[37] Vluggens A., Andreoletti P., Viswakarma N., Jia Y., Matsumoto K., Kulik W., Khan M., Huang J., Guo D., Yu S. (2010). Reversal of mouse Acyl-CoA oxidase 1 (ACOX1) null phenotype by human ACOX1b isoform. Lab. Investig..

[38] Riss T.L., Moravec R.A., Niles A.L., Duellman S., Benink H.A., Worzella T.J., Minor L., Sittampalam G.S., Coussens N.P., Nelson H., Arkin M., Auld D., Austin C., Bejcek B., Glicksman M., Inglese J., Iversen P.W. (2004). Cell viability assays. Assay Guidance Manual.

[39] Min S., More S.V., Park J.Y., Jeon S.B., Park S.Y., Park E.J., Yoon S.H., Choi D.K. (2014). EOP, a newly synthesized ethyl pyruvate derivative, attenuates the production of inflammatory mediators via p38, ERK and NF-κB pathways in lipopolysaccharide-activated BV-2 microglial cells. Molecules.

[40] Liu B., Wang K., Gao H.M., Mandavilli B., Wang J.Y., Hong J.S. (2001). Molecular consequences of activated microglia in the brain: Overactivation induces apoptosis. J. Neurochem..

[41] Jung D.Y., Lee H., Jung B.Y., Ock J., Lee M.S., Lee W.H., Suk K. (2005). TLR4, but not TLR2, signals autoregulatory apoptosis of cultured microglia: A critical role of IFN-β as a decision maker. J. Immunol..

[42] Song F., Zeng K., Liao L., Yu Q., Tu P., Wang X. (2016). Schizandrin A Inhibits Microglia-Mediated Neuroninflammation through Inhibiting TRAF6-NF-κB and Jak2-Stat3 Signaling Pathways. PLoS ONE.

[43] Wang Y., Gao H., Zhang W., Zhang W., Fang L. (2015). Thymoquinone inhibits lipopolysaccharide-induced inflammatory mediators in BV2 microglial cells. Int. Immunopharmacol..

[44] Khan M., Pahan K., Singh A.K., Singh I. (1998). Cytokine-induced accumulation of very long-chain fatty acids in rat C6 glial cells: Implication for X-adrenoleukodystrophy. J. Neurochem..

[45] Paintlia M.K., Paintlia A.S., Contreras M.A., Singh I., Singh A.K. (2008). Lipopolysaccharide-induced peroxisomal dysfunction exacerbates cerebral white matter injury: Attenuation by *N*-acetyl cysteine. Exp. Neurol..

[46] Khan M., Contreras M., Singh I. (2000). Endotoxin-induced alterations of lipid and fatty acid compositions in rat liver peroxisomes. J. Endotoxin Res..

[47] El Hajj H.I., Vluggens A., Andreoletti P., Ragot K., Mandard S., Kersten S., Waterham H.R., Lizard G., Wanders R.J., Reddy J.K. (2012). The inflammatory response in acyl-CoA oxidase 1 deficiency (pseudoneonatal adrenoleukodystrophy). Endocrinology.

[48] Oh P.S., Lim K.T. (2006). Glycoprotein (90 kDa) isolated from Opuntia ficus-indica var. saboten MAKINO lowers plasma lipid level through scavenging of intracellular radicals in Triton WR-1339-induced mice. Biol. Pharm. Bull..

[49] Lee M.H., Kim J.Y., Yoon J.H., Lim H.J., Kim T.H., Jin C., Kwak W.J., Han C.K., Ryu J.H. (2006). Inhibition of nitric oxide synthase expression in activated microglia and peroxynitrite scavenging activity by *Opuntia ficus indica* var. saboten. Phytother. Res..

[50] Kim J.H., Park S.M., Ha H.J., Moon C.J., Shin T.K., Kim J.M., Lee N.H., Kim H.C., Jang K.J., Wie M.B. (2006). Opuntia ficus-indica attenuates neuronal injury in in vitro and in vivo models of cerebral ischemia. J. Ethnopharmacol..

